# Molecular and Glycosylation Pathways in Osteosarcoma: Tumor Microenvironment and Emerging Strategies Toward Personalized Oncology

**DOI:** 10.3390/cimb47080629

**Published:** 2025-08-07

**Authors:** Georgian Longin Iacobescu, Antonio-Daniel Corlatescu, Horia Petre Costin, Razvan Spiridonica, Mihnea-Ioan-Gabriel Popa, Catalin Cirstoiu

**Affiliations:** 1 Faculty of General Medicine, Department of Orthopaedics, “Carol Davila” University of Medicine and Pharmacy, 050474 Bucharest, Romaniahoria-petre.costin0720@stud.umfcd.ro (H.P.C.);; 2 University Emergency Hospital Bucharest, 050098 Bucharest, Romania

**Keywords:** osteosarcoma, tumor microenvironment, cancer stem cells, glycosylation, immunotherapy, biomarkers

## Abstract

Osteosarcoma (OS) is the most common primary bone malignancy in children and adolescents, which is also considered an aggressive disease due to its rapid growth rate, ability to metastasize early, and complex and heterogeneous tumor microenvironment (TME). Although we are developing improved surgical and chemotherapeutic approaches, the presence of metastatic or recurrent disease is still detrimental to the patient’s outcome. Major advances in understanding the molecular mechanisms of OS are needed to substantially improve outcomes for patients being treated for OS. This review integrates new data on the molecular biology, pathophysiology, and immune landscape of OS, as well as introducing salient areas of tumorigenesis underpinning these findings, such as chromothripsis; kataegis; cancer stem cell dynamics; and updated genetic, epigenetic, and glycosylation modifiers. In addition, we review promising biomarkers, diagnostic platforms, and treatments, including immunotherapy, targeted small molecule inhibitors, and nanomedicine. Using genomic techniques, we have defined OS for its significant genomic instability due to TP53 and RB1 mutations, chromosomal rearrangements, and aberrant glycosylation. The TME is also characterized as immunosuppressive and populated by tumor-associated macrophages, myeloid-derived suppressor cells, and regulatory T cells, ultimately inhibiting immune checkpoint inhibitors. Emerging fields such as glycomics and epigenetics, as well as stem cell biology, have defined promising biomarkers and targets. Preclinical studies have identified that glycan-directed CAR therapies could be possible, as well as metabolic inhibitors and 3D tumor models, which presented some preclinical success and could allow for tumoral specificity and enhanced efficacy. OS is a biologically and clinically complex disease; however, advances in exploring the molecular and immunologic landscape of OS present new opportunities in biomarkers and the development of new treatment options with adjunctive care. Successful treatments in the future will require personalized, multi-targeted approaches to account for tumor heterogeneity and immune evasion. This will help us turn the corner in providing improved outcomes for patients with this resilient malignancy.

## 1. Introduction

Osteosarcoma (OS) is the most frequently diagnosed primary bone cancer that occurs in children and adolescents. It mainly forms in the metaphysis and diaphysis of the long bones by forming osteoid and immature bone [1]. Osteosarcoma represents approximately 20% of all bone cancers and generally presents as an aggressive tumor that exhibits local invasiveness and a relatively high risk of early pulmonary metastases. This aggressive behavior of osteosarcoma leads to rapid bone destruction accompanied by significant functional impairment [2]. Current management of osteosarcoma (primarily consisting of surgical resection alongside neoadjuvant chemotherapy agents such as doxorubicin, cisplatin, methotrexate, and ifosfamide) may provide benefits in cases of localized disease; however, the prognosis remains mostly limited if the osteosarcoma is distinguished as metastatic or recurrent. The significant difference in the five-year survival rate for localized osteosarcoma (greater than 78%) versus the percentage of long-term survivors with metastatic or recurrent osteosarcoma (25%) demands newfound innovative and effective therapies [3]. Although the MAP regimen, consisting of high-dose methotrexate, doxorubicin, and cisplatin, revolutionized treatment decades ago, survival rates have since remained relatively stagnant, largely due to the tumor’s genetic heterogeneity, complex microenvironment, and a lack of distinct targetable antigens. As such, meaningful progress will likely require a departure from intensified cytotoxic regimens toward modern strategies such as tyrosine kinase inhibitors, immunotherapies, and agents targeting tumor-specific cell surface markers [4].

Given the heterogeneity, local progression, and treatment resistance of osteosarcoma, more attention has been directed toward the osteosarcoma tumor microenvironment (TME), which contains a diverse mixture of osteosarcoma cells and adjacent stromal, vascular, and immune cells embedded in a mineralized extracellular matrix [5]. The unique microenvironment permits these cellular interactions that modulate a variety of different processes such as invasion, apoptosis, angiogenesis, metastasis, and response to therapy [6]. It is also important to highlight the immune features of the TME that are associated with the immunosuppressive elements, which include the following: tumor-associated macrophages (TAMs); neutrophils (TANs); myeloid-derived suppressor cells (MDSCs); regulatory T cells (Tregs); and key immunoregulatory molecules such as PD-1, IL-10, TGF-β, vascular endothelial growth factor (VEGF), and STAT3. Together, these elements contribute to an environment that protects the tumor from an effective immune response [7,8,9,10]. Although exogenous immunotherapy targeting PD-1 [11] has been trialed in osteosarcoma, there have been minimal clinical responses seen, indicating the challenges of how patients with osteosarcoma are limited to the immunosuppressive TME [12,13]. Therefore, to discover promising therapeutic targets from osteosarcoma, a better understanding of the immune–stromal–tumor interdependencies and tumor heterogeneity is needed to improve the effectiveness of immunotherapeutic opportunities [14].

For high-grade osteosarcoma, the conventional treatment approach consists of neoadjuvant chemotherapy followed by surgical resection and adjuvant chemotherapy. In contrast, low-grade osteosarcoma is typically managed with wide surgical excision alone, with chemotherapy reserved for cases exhibiting dedifferentiation or recurrence [15,16]. Distant metastases occurring during the course of treatment remain a major prognostic factor with respect to survival in initially non-metastatic patients. This further emphasizes the importance of chemotherapy in eliminating micrometastases. The current recommendation for pediatric and adolescent patients is that neoadjuvant chemotherapy should consist of the MAP regimen, while adult patients are recommended to receive combination doxorubicin plus cisplatin. The PEI regimen of cisplatin, ifosfamide, and etoposide is also used in patients of both age groups. A favorable histopathologic response of ≥90% necrosis following neoadjuvant chemotherapy has been associated with increased survival in a variety of post-operative chemotherapy regimens [17].

We adopt a proteomics-centred vantage because it furnishes the most direct bridge between gene transcription and the functional protein alterations, chiefly aberrant glycosylation, that underlie osteosarcoma pathogenesis. Recent proteomic investigations have illuminated a rich repertoire of glycosylation-based biomarkers and actionable therapeutic targets, insights that are still less developed in metabolomic or non-proteomic post-translational studies. While these complementary disciplines are acknowledged for context, our emphasis on proteomics reflects its singular translational promise.

## 2. Biology and Pathophysiology of Osteosarcoma

Osteosarcoma is a heterogeneous malignancy that contains many different cellular subpopulations that vary by morphology, gene expression, metabolism, and metastatic ability. To our current understanding of osteosarcoma, osteosarcoma is influenced by its heterogeneous bone microenvironment, containing osteoblasts, osteoclasts, osteocytes, mesenchymal stem cells (MSCs), immune cells, and a dynamic mineralized extracellular matrix. Heterogeneity is a common occurrence within cancer research and has been observed through conventional transcriptomics with marginal improvement [18]. On the other hand, in a study in which they utilized single-cell RNA sequencing (scRNA-seq) for their analysis, they identified 11 unique cell clusters within osteosarcoma [19]. These unique clusters included different cellular types such as osteoblastic cells, tumor-infiltrating lymphocytes (TILs), chondroblastic cells, MSCs, and endothelial cells that varied depending on whether the tumor was primary, recurrent, or ultimately metastatic [20]. Another study reported variations in the cellular composition based on the stage of tumor progression in their scRNA-seq study, with a higher abundance of TILs and MSCs in metastatic OS [21]. Increased immune cell infiltration, particularly (TILs), is generally associated with improved clinical outcomes in many malignancies. However, in osteosarcoma, the prognostic significance of TILs appears to be more complex and context-dependent, reflecting the heterogeneous and often immunosuppressive nature of its tumor microenvironment [19,20].

The bone microenvironment modulates the osteosarcoma activity through different signaling pathways related to bone remodeling and growth factors. Moreover, as bone undergoes degradation, many different growth factors (e.g., TGF-β and FGFs) are released from the matrix that, through signaling, sustains or enhances tumor proliferation as a part of a feedback loop. An example of growth factors is TGF-β1, which is prominent in the osteosarcoma bone microenvironment and is primarily involved in tumor growth and metastasis. Further, FGFs play a huge role in pre-osteoblast proliferation (FGF2) and osteoblast maturation (FGF18) [22].

Recent findings underscore the role of osteosarcoma-derived extracellular vesicles (EVs) in promoting osteoclastogenesis and bone resorption. EVs from highly metastatic cell lines were shown to enhance osteoclast differentiation, contributing to the tumor’s osteolytic behavior. Notably, blocking EV secretion impaired osteoclast formation, highlighting the potential of targeting EV-mediated signaling as a therapeutic strategy to disrupt tumor–bone microenvironment interactions [23].

Osteosarcoma is characterized by considerable genomic instability with frequent loss of heterozygosity, expansive gene amplifications, deletions, and chromosomal rearrangements. An important feature of genomic instability is chromothripsis that can be observed in approximately 77% of osteosarcoma, that generates massive fragmentation and reassembly of the genome often leading to amplifications (e.g., CDK4, MDM2), gains (RICTOR, TERT), and disruptions (TP53, NF1) of chromosomal loci 5, 6, 12, 13, 14, and 17 [24]. Kataegis is another hallmark genomic alteration found in as many as 85% of osteosarcoma samples in the form of hypermutation clusters; however, kataegis is rare near TP53 or ATRX [25,26]. Collectively, structural alterations can underlie the aggressiveness of osteosarcoma, as well as highlighting the heterogeneity of osteosarcoma that accounts for the refusal to respond to standard therapies.

On a molecular level, the role of driver gene mutations is fundamental in tumorigenesis. With frequent alterations of TP53 and RB1 seen in pediatric cases, other highly implicated cancer-causing genes are NOTCH1, MYC, and FOS, which have been shown to strongly initiate tumor formation [27,28]. The “driver” mutations cooperate with “synergistic” gene changes, as is the case with “driver” gene mutations in PTEN, JUN, or TWIST, which are less able to cause cancer alone but likely make it worse (these altered genes likely contribute to progression on collaborative partnerships with “driver” mutations) [29]. In the analysis of osteosarcoma, transcriptomics classified over 3000 differentially expressed genes, expressing specific markers with high expression, such as MMP14, COL11A1, and PKM2, all with invasive, matrix remodeling, and metabolic functions [30]. In addition, several biomarkers are associated with tumor-promoting signaling pathways and adverse clinical outcomes; for instance, cathepsin D, miR-421, and HMGB1 are frequently overexpressed in osteosarcoma and linked to enhanced tumor progression. Conversely, reduced expression of tumor suppressors such as FBXW7 has been correlated with increased cellular proliferation and aggressiveness [31,32,33].

## 3. Glycosylation and Tumorigenesis

Abnormal glycosylation is increasingly thought to be a hallmark of cancer, which is also the case in osteosarcoma, where modified glycan structures found on tumor cell surfaces have critical roles in driving cellular behavior, immune evasion, and metastasis. In osteosarcoma, tumor cells commonly have increased branching of N-glycans due to the activity of the enzyme MGAT5, which promotes prolonged growth factor signaling and an increase in metastatic potential. While fucosyltransferases such as FUT8 are generally implicated in promoting tumor progression through core fucosylation of N-glycans, recent findings suggest a more complex and possibly inverse role in osteosarcoma. In particular, a recent relevant study demonstrated that FUT8 downregulation in osteosarcoma cells enhanced metastasis via upregulation of L1CAM, indicating a potential tumor-suppressive function in this context [34]. These observations highlight the importance of tumor-specific glycosylation patterns, which are not random but are likely selected during tumor evolution to support survival, immune evasion, and metastatic potential. Moreover, the TME in osteosarcoma, rich in stromal and immune components, may further influence the impact of glycan-mediated signaling, contributing to therapeutic resistance and intertumoral heterogeneity [35,36].

Changes in O-glycosylation, particularly altered expression of truncated structures such as Tn and sialyl-Tn (STn) antigens, may allow osteosarcoma cells to detach while surviving immune detection, and disseminate metastatically. Mucins that carry O-glycosylation in these forms may act as antiadhesive biomolecules, preventing cell-cell interactions so that tumor cells can dislocate and thereby promote their own metastatic spread. Tumor-associated sialoglycans can facilitate vascular adhesion and extravasation by interacting with selectins on endothelial cells and platelets, and on endothelial cells during the initial stages of metastasis [37]. It can also be expected that sialylated structures can bind, inhibiting Siglecs on immune cells as a mechanism of local immune suppression. Although these glycosylation-driven mechanisms were previously thought to only facilitate and support tumor growth and immune escape/evasion, they present potential biomarkers/therapeutic targets in osteosarcoma. Identifying glycan expression patterns as well as understanding these biological mechanisms may represent the possibility of new diagnostic approaches and the development of glycan-targeted therapies in the future with this highly aggressive cancer [38].

Cancer stem cells (CSCs) constitute a small but highly plastic sub-fraction of osteosarcoma that drives tumour heterogeneity, relapse, and metastatic spread; they are typically enriched in side-population or ALDH-bright fractions and display elevated ABC-transporter activity together with surface markers such as CD133 and CD44 [39]. Experimental reprogramming of conventional osteosarcoma lines with the pluripotency factors OCT3/4, KLF4, and SOX2 confirms that these transcriptional circuits are sufficient to confer a CSC phenotype, characterised by up-regulation of CD24, CD26, and CD133, enhanced sphere formation, osteogenic capacity, migration, and broad chemoresistance [40]. Mechanistically, sustained stemness hinges on post-translational stabilisation of SOX2: AKT physically associates with and phosphorylates SOX2 at Thr116, shielding it from UBR5- and STUB1-mediated ubiquitin-proteasomal degradation; pharmacological inhibition of AKT therefore destabilises SOX2, suppresses CSC traits, and restores cisplatin sensitivity in patient-derived xenografts [41]. Beyond kinase blockade, a second therapeutic avenue involves nano-engineering; smart polymeric, lipidic, or inorganic nanocarriers functionalised with antibodies or ligands against CSC-specific markers enable preferential drug deposition within the stem-cell niche, simultaneously limiting off-target toxicity and overcoming efflux-mediated resistance [42].

Recently, the notion that a subset of osteosarcoma cells (osteosarcoma stem cells (OS-CSCs)) is primarily responsible for initiating, progressing, and relapsing tumors has been supported with extensive work being published. OS-CSCs can present stem-like properties such as self-renewal, pluripotency, and very high tumorigenicity, and presumably originate from transformed mesenchymal stem cells (MSCs) or osteoblastic progenitor cells that have been influenced by epigenetic, genetic, and microenvironmental alterations [43,44]. In vivo studies have demonstrated that the concomitant loss of certain tumor suppressors like TP53 and RB1 in MSC can lead to tumor formation that would resemble an OS, while transforming osteoblast precursors would suggest a similar outcome [44,45]. OS-CSCs are nurtured by the tumor microenvironment, and their interactions, notably with CAFs and MSCs, promote the dedifferentiation and preservation of stemness through paracrine signalling and vesicular pathways generally associated with TGF-β [46,47,48]. These tumor-initiating cells contribute to tumor heterogeneity, resistance to therapy, and metastases, and in part, they are able to do this by creating unique subclones and manipulating the protective niche that surrounds them [49,50]. Importantly, OS-CSCs are known to resist hypoxia and the effects of chemotherapy and radiotherapy, rendering them direct and significant targets for future therapeutic strategies [50,51]. The recognition of OS-CSCs and their environment facilitated advances in 3D tissue-engineered models, because it better recreates the native tumor and will activate critical pathways, including MAPK, PI3K, and TGF-β/SMAD pivotal to the pathogenesis of osteosarcoma [52,53].

Single-cell RNA-seq, multiplex immunofluorescence, and spatial transcriptomics now show that OS lesions are dominated by myeloid lineages rather than by lymphocytes. Primary tumours and, even more, lung metastases contain three hierarchically related macrophage states: pro-inflammatory M1, immunosuppressive M2, and a metastasis-enriched FABP4⁺ programme that fuels cancer-stem-cell renewal and drug resistance [54]. CD14^+^HLA-DR^−^ myeloid-derived suppressor cells (MDSCs) accumulate in the same niches; physical contacts between MDSCs and PD-1^+^ T cells spatially mark the poorest-prognosis metastases, while dendritic cells are skewed toward a LAMP3^+^CCR7^+^ “mregDC” phenotype that secretes CCL17/19/22 to recruit TIGIT^+^/TIM-3^+^ regulatory T cells [2]. These suppressive circuits are reinforced by cancer-cell evasion strategies (loss of MHC-I, up-regulation of the “don’t-eat-me” signal CD24, and induction of PD-L1), creating immune-cold tumour cores that exclude cytotoxic CD8^+^ cells and natural-killer subsets [55]. A newly proposed “osteosarcoma spatial score” that captures the ratio of immune–tumour juxtaposition to immune-desert areas stratifies five-year survival more accurately than PD-L1 staining and is being built into biomarker-driven trials [56].

Therapeutically, these insights are steering combination strategies that go beyond checkpoint blockade. Preclinical work shows that all-trans retinoic acid or MerTK inhibition can repolarise M2 and FABP4^+^ macrophages to an M1 state, while CSF1R and CXCR2 antagonists curb MDSC recruitment [54]. Oncolytic viruses armed with CXCL9/10 genes reopen immune-cold nests and synergise with anti-PD-1 in patient-derived xenografts [56]; GD2- or B7-H3-targeted CAR-NK and CAR-T cells are being engineered with IL-15 payloads to overcome myeloid barricades [55]. Finally, pharmacological dismantling of neutrophil extracellular traps or induction of tertiary lymphoid structures with STING agonists further augments T-cell trafficking and improves survival in mouse models [57]. Together, a myeloid-centred view of the OS tumour micro-environment, coupled with spatial biomarkers, is redefining clinical trial design and holds promise for the next generation of precision immunotherapies.

## 4. Genetic and Epigenetic Changes

Recent technological developments in whole genome sequencing have illustrated an incredible complexity in the osteosarcoma genome and have highlighted chromothripsis as an important driver of osteosarcoma evolution. In contrast to other tumors where chromothripsis is a more static process occurring early in evolution from the cancer cell, chromothripsis in osteosarcoma appears to be a dynamic process contributing to clonal diversity and intra-tumor heterogeneity [24,58]. A new mechanism termed loss-translocation-amplification (LTA) chromothripsis that has been described in approximately 50% of high-grade osteosarcomas in which two pathways converge to simultaneously inactivate TP53, and amplify multiple oncogenes through successive break-fusion-bridge cycles, makes osteosarcoma represent very complex chromosomal rearrangements in human cancer. This not only relates this chromothripsis pathway specifically to osteosarcoma, but also reinforces the concept of structural genomic alterations as being central to the pathogenesis and evolution of osteosarcoma [59].

A recent multicenter study reported that genetic variation near the GRB10 gene on human chromosome 7p12.1 may represent a potentially novel risk factor for osteosarcoma and implicates conserved growth-regulatory pathways shared among humans and canines. This genome-wide association study used data from >1000 pediatric osteosarcoma patient cases and >3000 controls and depicted the first genetic variation related to osteosarcoma. The association was generally driven by an SNP (rs17454681) located upstream of the GRB10 gene. The SNP resides in a regulatory region that possesses histone modifications suggestive of enhancer and promoter activity in mesenchymal stem cells and osteoblasts, the very cell types implicated in the development of osteosarcoma, representing a novel and labeled genetic risk for osteosarcoma. Functional studies indicate that GRB10, an imprinted gene that has been characterized as an inhibitor of tyrosine kinase receptors such as IGF1R, PDGFRB, and EGFR, may also be important in both normal bone development and malignant transformation. The research demonstrates the efficacy of comparative oncology in identifying a genetic risk factor for osteosarcoma and also emphasizes that osteosarcoma predisposition is a complex interaction between growth signaling, imprinting, and tumor susceptibility [60].

Epigenetic changes have been identified as critical components of the pathobiology of osteosarcoma (OS) that illuminate pathways of disease causation that are not easily attributed to mutations. According to Twenhafel et al. [61], DNA methylation is the most extensively studied epigenetic modification in osteosarcoma. Aberrant hypermethylation of promoter regions can lead to the transcriptional silencing of tumor suppressor genes as well as non-coding RNAs, including miR-149 and miR-195. These microRNAs normally function to inhibit tumor cell proliferation, invasion, and metastasis; however, their methylation-induced downregulation removes this inhibitory control, thereby contributing to tumor progression. Increased expression of DNA methyltransferases (DNMTs) in OS resulted in genes with immune-regulatory functions, such as CXCL12, being suppressed, enabling immune evasion and facilitating the spread of metastasis. Other studies have implicated histone modifications, especially KDM6A/B, regulating demethylation of H3K27me3, as important regulators of metastasis and resistance to chemotherapy, and that the m6A RNA methylation resulted in METTL3 overexpression that favored tumor-promoting gene expression such as DRG1 and ATAD2 [61].

In addition to DNA and histones, non-coding RNAs (ncRNAs) such as long non-coding (lnc) RNA and circular (circ) RNA are also linked to epigenetic regulation in OS. Long non-coding RNA (LncRNAs), such as THAP9-AS1 and HOTAIR, can recruit DNMTs to gene promoters, and alter the methylation patterns that promote tumor cell proliferation and migration [62]. Circular RNA (CircRNAs) such as CircECE1 is shown to promote aerobic glycolysis and metastasis by activating c-Myc, and circMTO1 inhibits tumor progression via acting on the miR-630/KLF6 axis. These studies exhibit the significant complexity of epigenetic networks in OS and the promising clinical relevance of epigenetics in OS as diagnostic biomarkers and therapeutic targets [63] (Table 1).

## 5. Glycan Biomarkers

Glycan biomarkers are emerging as an important class of molecular biomarkers for the diagnosis and follow-up of multiple cancers, arising from the inherently complex stereochemistry of glycan protein glycosylation changes in a disease state. N-glycans in particular play an important role in biological function, including but not limited to cell-cell communication, immune response, and protein stability, with considerable glycan changes during cancer progression [64]. Recent advances in glycomics, notably mass spectrometry-related applications, allow researchers, clinicians, and industry partners to record glycan changes with high throughput and sensitive analysis in serum and plasma sources. Specifically, however, mass spectrometry-based platforms have documented glycan compositional changes in other types of cancers internalizing in the aegis of previously disclosed candidate diagnostic/prognostic biomarkers (increased sialylation and fucosylation or increased high mannose glycans). Larger studies have also reported cancer-specific glycosylation signatures (greater sialyl Lewis × structures in breast and ovarian cancers or the bending and shift of tri- and tetra-antennary glycans in gastric and lung malignancies) [65].

The future for biomarker discovery, at multiple levels of glycome complexity (the types, or classes, of glycan) and non-template driven glycan synthesis (from monosaccharide compositions to protein-and site-specific glycosylation) looks promising. Current analytic workflows have been as simple as profiling and characterizing native glycans using MALDI-FTICR-MS, complete with isomer-specific separation of glycans by porous graphitic carbon liquid chromatography coupled with time-of-flight mass spectrometry [66]. These emerging technologies not only have the potential to significantly improve specificity in glycan biomarker discovery, but also develop the uncommon definition of glycan libraries and correlation networks, and thereby provide a systemic manner of glycosylation changes in cancer. Regardless, we still need to consider the clinical translatability; the importance of large validation studies; the use of QC methods; using standardization methods pre-analytically and post-analytically; and using bioinformatics methods to visualize, integrate, and analyze glycomics data within existing clinical interrogation scales and models of systems biology. Regardless, the field offers significant opportunities for the development of sensitive and specific glycan-based non-invasive diagnostics [67].

Aberrant glycosylation is a current hallmark of cancer and is fundamental to the diagnosis and prognosis of numerous tumors. Considerable tumor-associated glycan patterns, including truncated O-glycans (Tn, T, and sialyl-Tn antigens), increased sialylation, and increased fucosylation are commonly found in breast, gastric, pancreatic, and ovarian tumors, which often appear due to dysregulated glycosyltransferase activity associated with tumor progression, metastasis, and immune evasion. Sialylated Lewis antigens (sLe^x and sLe^a) are often overexpressed in various cancers, which indicates a poor prognosis, whereas the core fucosylated glycoform of alpha-fetoprotein (AFP-L3) is a more specific biomarker (than serum AFP) for hepatocellular carcinoma. Fucosylated haptoglobin has also been shown to have a better diagnostic sensitivity than other traditional markers in pancreatic cancer studies, supporting the clinical relevance of glycan-based biomarkers.

Relevant advancements have been made in the field of glycomics through a variety of methods and different types of analyses, including immunochemical-based detection, lectin-based detection, and mass spectrometry (MS) based methodologies. Mass spectrometry stands apart because it is often the method of choice for glycan structural analysis and site-specific and detailed glycan profiling. Mass spectrometry-based glycomic methods such as matrix-assisted laser desorption/ionization time-of-flight MS (MALDI-TOF) and electrospray ionization MS (ESI-MS) in both top-down and bottom-up glycomic fashion have facilitated high-throughput identification of glycoproteins and glycoproteins associated with cancer [68,69]. The increasing use of complementary methods, such as lectin microarrays and metabolic labeling, advances increased sensitivity and specificity by focusing on glycan motifs on the cell surface. These complementary methods provide tools not only for the identification of novel biomarkers but will also foster studies that will help elucidate glycan-mediated mechanisms in tumor biology to allow the introduction of glycan biomarkers into predictive, preventive, and personalized (3P) medicine strategies [70].

Biomarkers in osteosarcoma represent a clear opportunity to improve prognostic estimates and personalize treatment, particularly in patients who show limited response to conventional chemotherapy. Although RB1 and TP53 are frequently mutated and represent important foundations of osteosarcoma tumorigenesis, they have limited predictive potential for moderating response to therapy. In general, overexpression of distinctly characterized proteins [like glutathione S-transferase P1 (GSTP1) and chemokine receptor CXCR4] and cytoskeletal linker proteins has been correlated with poor outcome, distant metastases, and higher rates of metastatic spread [71,72,73]. Recent advances in high-throughput molecular profiling technologies, such as RNA-Seq and circRNA microarrays, have enhanced the evaluation of complex molecular expression patterns, enabling the identification of biomarkers relevant to osteosarcoma progression, metastasis, and treatment resistance. Circular RNAs (circRNAs), a novel class of non-coding RNAs with remarkable stability due to their closed-loop structure, have emerged as significant players in cancer biology. This updated meta-analysis identified 58 dysregulated circRNAs (52 upregulated and 6 downregulated) across 226 studies, highlighting their potential as diagnostic and prognostic biomarkers in osteosarcoma. Notably, circRNAs such as hsa_circ_0001821 and hsa_circ_0005721 showed consistent dysregulation across both tissue and blood samples, with hsa_circ_0005721 showing promise as a circulating biomarker. Although no single circRNA has yet been fully integrated into clinical workflows, the study demonstrated high diagnostic accuracy (AUC = 0.87), suggesting circRNAs’ potential to stratify responders versus non-responders and to guide personalized treatment strategies. Bioinformatics analyses also revealed that dysregulated circRNAs influence key cancer pathways, including VEGF, FoxO, and cell cycle regulation, supporting their role in osteosarcoma pathogenesis and therapeutic targeting [74].

It has recently been reported that the ligands recognized by C-type lectin CD301 (CLEC10A/MGL), which bind with the Tn and sialyl-Tn antigens, were identified as moderately to strongly expressed in approximately 26% of osteosarcoma tissue samples. The study used staining with recombinant CD301 to identify expression, and primary tumors were clearly distinguishable as positive for CD301 when adjacent normal bone and marrow tissues were only weakly bound. In contrast to other studies in some carcinomas, CD301 ligands with respect to survival in osteosarcoma were negative, which suggests that ligands representing a unique molecular signature may provide a basis for targeted therapy exploitability. CD301 is a lectin with clear evidence of binding specifically to these glycans, and its potential usefulness for disease classification or as immunotherapeutic agents, especially in the hypo-glycoengineered context of osteosarcoma, employing glyco-engineered treatments, strongly warrants further research [64].

## 6. Therapeutic Implications and Challenges

Despite significant effort over the last couple of decades, osteosarcoma treatment has been mainly stagnant, with surgical resection and high-dose chemotherapy maintaining the standard of care. The long-term patient outcomes remain poor, especially when evaluating patients who are metastatic or have relapsed, with a 5-year survival rate below 29%. One major impediment to the progressive development of effective treatment options lies in the underperformance of poorly characterized preclinical models, which fail to replicate human cancer complexity and inhibit the translation success of treatments that appear otherwise promising. The development of effective gene therapies or small molecule inhibitors is particularly challenging due to the high genetic heterogeneity and adaptive capacity of osteosarcoma cells, which often leads to therapeutic resistance and disease progression [75,76]. Other potential therapeutic pathways such as cancer vaccines, monoclonal antibodies, or other immunotherapeutics, including adoptive cell therapy (CAR T Cells, and tumor-infiltrating lymphocytes), have been demonstrated as safe and effective in preclinical studies but the tumor microenvironment represented in osteosarcoma, exemplified by its immunosuppressive, antigenically heterogeneous properties, represent a significant challenge in a clinical context. Future progress systems incorporating multiple targets based on combination therapies, optimizing immunotherapeutics, and personalizing treatments based on the molecular profile of tumors will ultimately be required for improved patient outcomes [77,78].

Notable studies include Liao et al. [79], which showed that the tyrosine kinase inhibitor sulfatinib targets FGFR1, CSF1R, VEGFR 1–3, and inhibits tumor growth and invasion while diminishing immunosuppressive populations in the TME. Another relevant study determined that gossypetin induced apoptosis in osteosarcoma cells through activation of Bax and caspase-3, while simultaneously reducing pro-inflammatory cytokine levels [80]. A nanomedicine-based approach [81], a ROS-and esterase-responsive formulation combining diCTX and diDHA, was designed to enhance selective cytotoxicity in osteosarcoma cells. Additional studies have described multiple molecular inhibitors with therapeutic potential, including soy isoflavones [82], which suppress AKT/mTOR signaling, and psoralidin [83], which interferes with FAK and PI3K/AKT signaling pathways. Furthermore, engineered hepatic circuits have been developed for site-specific delivery of VEGFR2 siRNA, offering a gene-targeted anti-angiogenic strategy. Another review [84] stated that the tumor progression driver, FGD1, was a novel target via phosphatase and tensin homolog (PTEN) inhibition. The combination of the peroxisome proliferator-activated receptor gamma (PPARγ) agonist pioglitazone and cisplatin for osteosarcoma treatment has been effective [85]. Clinically, glembatumumab vedotin has been demonstrated to have antitumor activity in recurrent or refractory osteosarcoma patients, and a combination of lenvatinib, etoposide, and ifosfamide has also demonstrated activity in patients with recurrent or refractory osteosarcoma [86].

The discovery of CD301 glycan ligands within osteosarcoma induced efforts to exploit them for targeted immunotherapy. A groundbreaking method utilizes natural killer (NK) cells engineered with a CD301-based chimeric antigen receptor (CAR), which induced specific cytotoxicity against ligand-positive osteosarcoma cell lines. In addition to enhanced tumor cell killing, these CD301-CAR NK92 cells also produced a downpour of interferon-gamma secretion and degranulation, indicating potent antitumor activity. Clinical translation is implicated by the possible off-tumor effects, since some normal tissues express CD301 ligands, particularly within the gastrointestinal tract. Further, the partial efficacy of immune checkpoint blockade (such as with TIGIT inhibition) to increase CTL proliferation of CAR NK92 cells in this space points towards combined strategies [64].

A recent understanding of the molecular complexity of osteosarcoma has slowly led to an interest in more targeted and individualized therapies. Metabolic reprogramming is one such strategy, notably with the targeting of key enzymes like PHGDH in the de novo serine biosynthesis pathway. The inhibition of PHGDH reduces tumor cell proliferation; however, it invokes a pro-survival compensatory pathway through mTORC1 activation [80]. Exciting preclinical work demonstrated that dual inhibition of PHGDH-mTORC1 produced a synergistic cytotoxicity across osteosarcoma models. This new combination of metabolic therapy is unique, and the complexities of tumor metabolism and heterogeneity of osteosarcoma represent a significant challenge for the development of therapies. Studying combination strategies with multiple targets and biomarkers for guiding patient selection will be necessary [87,88,89].

Immune checkpoint inhibitors (ICIs) represent a novel approach for the treatment of osteosarcoma, as osteosarcoma tumor cells have relatively high levels of immune cell infiltration [90]. Antibodies targeting PD-1, PD-L1, and CTLA-4, either as monotherapy or in combination with Sorafenib or Doxorubicin as a means to increase T cell responses, are promising [91,92]. Similarly, in another study, thrombospondin-1 (TSP1) and signal transducer and activator of transcription 3 (STAT3) enhanced PD-L1; however, when neutralizing antibodies were tested, restored CD8^+^ T cells activity was observed [93]. Other developments include IDO inhibitors [94], modulation in conjunction with microRNAs, and combinations using ICIs with an oncolytic virus (OBP-502), which seems to provide additional immunogenic cell death and increased T cell infiltration as a bonus [95].

**Figure 1:**
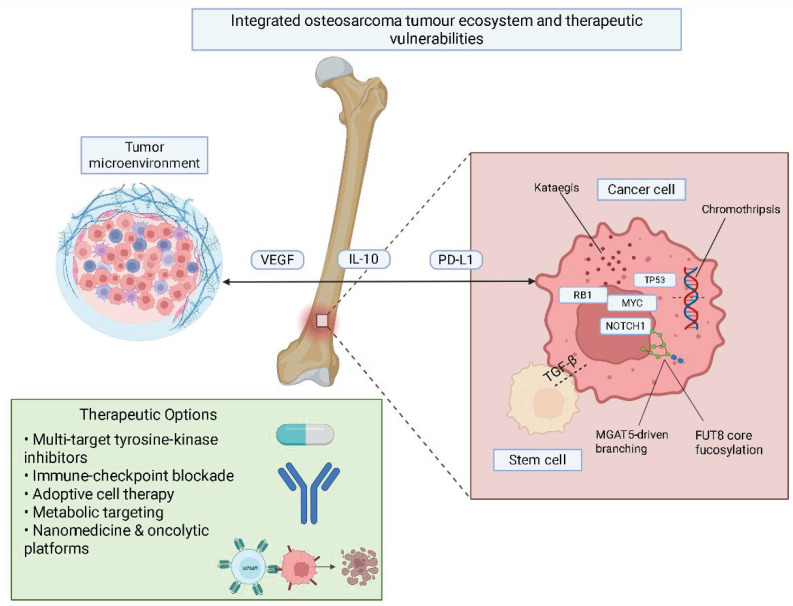
Integrated osteosarcoma tumour ecosystem and therapeutic vulnerabilities. This figure depicts the interaction between osteosarcoma cells, the TME, and therapeutic targets. Genetic instability (e.g., chromothripsis, kataegis) and key oncogenic alterations (TP53, RB1, MYC, NOTCH1) are shown alongside aberrant glycosylation (MGAT5, FUT8) and stem cell signaling (TGF-β). The immunosuppressive TME includes cytokines such as IL-10, VEGF, and PD-L1 that support tumor progression. Therapeutic options illustrated below target both tumor-intrinsic mechanisms and microenvironmental factors to disrupt osteosarcoma growth and resistance.

Resistance remains a challenge, likely based on PD-L1 over-expression and immune suppression. Better response rates have been observed when combining PD-1 blockade with chemo (or PD-1 blockade and PI3Kδ/γ inhibition) [96,97]. Nanocarrier systems that were developed to allow modulating PD-1/PD-L1 and autophagy demonstrated to improve CD8^+^ T cell activation [98]. In the tumor microenvironment, combinations of multi-points of blockade (PD-L1, CTLA-4, TIM3), combining radiation therapy with those ICIs, and immune modulation that uses sunitinib or L-arginine will again attempt to redefine the tumor microenvironment and redefine the immune response [99,100] Figure 1.

Although OS is traditionally regarded as radio-resistant, advances in high-precision particle therapy and the revitalised search for efficacious radiosensitisers are beginning to erode this therapeutic impasse. In anatomically constrained sites such as the craniofacial skeleton, where wide resection is often unfeasible, modern proton beam therapy and carbon-ion radiotherapy (CIRT) exploit the Bragg-peak phenomenon to confine dose deposition to the tumour bed, thereby sparing critical structures while delivering a higher relative biological effectiveness than conventional X-rays [101,102,103]. A recent systematic review demonstrated their particular suitability for sarcomas: the reduced integral dose spares growing tissues in younger patients, while carbon ions’ higher linear energy transfer yields more complex DNA damage and potentially greater tumour-cell kill than photons or protons alone [104]. These data strengthen the therapeutic rationale for integrating particle therapy, alone or in combination with radiosensitizers, into osteosarcoma treatment algorithms whenever surgical margins are questionable or critical organs are around the target volume. In another promising study, carbon minibeam radiation therapy (C-MBRT) was explored in a syngeneic murine osteosarcoma model and showed that delivering an average dose of only 20 Gy—of which 70% was a very low 1.5 Gy “valley” dose and <30% a 105 Gy “peak” dose—produced a tumour-growth delay comparable to that achieved with uniform high-dose conventional carbon-ion therapy while maintaining excellent normal-tissue tolerance. Despite the marked dose heterogeneity, C-MBRT elicited significant intratumoural CD8^+^ T-cell infiltration and reduced pulmonary metastasis burden, suggesting that spatially fractionated carbon-ion fields may trigger immunogenic mechanisms that transcend mere direct DNA damage [105].

While several innovative agents and delivery platforms have shown promise in preclinical osteosarcoma models, success in the clinic remains elusive. Reviews of current model systems indicate that both in vitro and even in vivo murine models often lack full immune and stromal components, producing barriers between preclinical success and clinical failure [106]. There are also additional limitations related to the absolute rarity and genetic heterogeneity of osteosarcoma that limit the design and statistical power of early-phase trials evaluating these agents [107]. Early human trials, even for studies targeting metastatic disease with strong preclinical rationales, successfully reproduce antitumor responses in unrealized ways, representing a significant translational bottleneck [108].

Future research into immune therapies for osteosarcoma needs an understanding of the immune factors in the immune-suppressed tumoral microenvironment (TME) and resistance to other therapies. Combination strategies with immune checkpoint inhibitors, cytotoxic, radiation, and targeted therapies can be useful to overcome some of the resistance and improve tumor-infiltrating lymphocyte resistance to the antitumor immune responses, and develop the T cell responses. Approaches like dual checkpoint inhibition (PD1 or CTLA4), and epigenetic modulatory inhibitors, or TME targeting agents to re-activate cytotoxic T lymphocytes or overwrite the immune landscape, a new area of cellular therapies also being under development in chimeric antigen receptor T cells (CAR-T) or chimeric antigen receptor natural killer cells (CAR-NK), and targeting high level of osteosarcoma relevant antigens like B7-H3 which have been extensively studied preclinically and are also under development along with immune adjuvants or cytokine support [109].

At the same time, the potential of new nanoscale carriers and future generations of delivery systems capable of co-encapsulating immunomodulatory drugs and tumor-specific inhibitors will provide a powerful platform to increase bioavailability and limit toxicity, while supporting immune responses. Additionally, there have been data utilizing redox-responsive micelles and polymeric nanoparticles to facilitate immunogenic cell death with co-delivery of TGF-β inhibitors and STING agonists directly into the TME. As biomaterial development and exosome-mimetic vesicle development continue in new directions, these approaches should help begin to address the issues of tumor heterogeneity and immune evasion, and provide a clearer approach towards personalized and improved immunotherapeutics for osteosarcoma [110].

## 7. Conclusions

Osteosarcoma continues to pose a significant clinical challenge due to its aggressive behavior, propensity for early metastasis, and pronounced intratumoral heterogeneity. While current treatment strategies have improved the outcomes associated with localized osteosarcoma primarily by surgeries and high-dose chemotherapy, patients with metastatic osteosarcoma and recurrent osteosarcoma have dismal prognoses. Better understanding of the molecular and cellular features of this tumor, including its unique TME, cancer stem cell dynamics (tumor heterogeneity and evolution of the metastatic clone), and epigenetic and glycomic alterations has opened new avenues to understanding its pathophysiology. This new knowledge has translated to more specific diagnostic biomarkers and potential new treatment strategies related to signaling, glycosylation, and/or immune evasion mechanisms.

Despite some recent advances with potential application in osteosarcoma in immunotherapy, targeted molecular inhibitors, and metabolic approaches, preclinical success has not yet translated to prolonged clinical efficacy as we try to adapt to the unique characteristics of this tumor. At this juncture, we need to make the shift to integrated, coordinated approaches to treatment that exploit immune modulation/biomarker-driven therapy/targeted patient-specific, multi-modal treatment strategies (ideally some combination treatment) to address the array of potential targets in osteosarcoma. Advances in our ability to use single-cell sequencing, 3D tumor models, and nanomedicine represent unique opportunities for the precision oncology paradigm we are all working to achieve. The way forward considers the clinical application of basic knowledge to design novel treatments tailored to improve efficacy, patient survival, and quality of life for patients with osteosarcoma.

## Figures and Tables

**Table 1:** Epigenetic drivers and emerging interventions in osteosarcoma.

Epigenetic Mechanism	Main Molecular Players (Examples)	Functional Consequence in Osteosarcoma	Clinical/Therapeutic Outlook
DNA-methylation gain	DNMT1/3A/3B; hypermethylation of miR-149, miR-195, CXCL12 [57]	Silencing of tumor suppressors and immune genes → promotes proliferation, invasion, and immune evasion [57]	DNMT inhibitors (decitabine, guadecitabine) [57]
Histone-mark loss (H3K27me3)	KDM6A/B erase H3K27me3 marks [57]	Enhances metastatic capacity and chemoresistance [57]	EZH2 agonists or KDM6A/B inhibitors under preclinical investigation [57]
m^6^A RNA methylation	METTL3 overexpression stabilizes DRG1, ATAD2 transcripts [57]	Drives glycolysis and cell-cycle progression [57]	METTL3 inhibitor (STM-2457) shows efficacy in preclinical OS models [57]
lncRNA-guided chromatin remodelling	lncRNAs THAP9-AS1, HOTAIR recruit DNMTs to gene promoters [58]	Fuels proliferation and migration [58]	Antisense oligonucleotides or CRISPR-based silencing in development [58]
circRNA networks	circECE1 (oncogenic); circMTO1 (tumor-suppressive) [59]	Regulate aerobic glycolysis, metastasis, and tumour suppression [59]	Potential use in liquid-biopsy diagnostics due to stability [59]
